# Energy cost differences between marathon runners and soccer players: Constant versus shuttle running

**DOI:** 10.3389/fphys.2023.1159228

**Published:** 2023-05-03

**Authors:** Johnny Padulo, Antonio Buglione, Alin Larion, Fabio Esposito, Christian Doria, Dražen Čular, Pietro Enrico di Prampero, Leonardo Alexandre Peyré-Tartaruga

**Affiliations:** ^1^ Department of Biomedical Sciences for Health, Università degli Studi di Milano, Milan, Italy; ^2^ Department of Human Sciences and Promotion of the Quality of Life, San Raffaele Roma Open University, Rome, Italy; ^3^ Faculty of Physical Education and Sport, Ovidius University of Constanta, Constanta, Romania; ^4^ IRCSS Galeazzi Orthopaedic Institute, Milan, Italy; ^5^ Faculty of Kinesiology, University of Split, Split, Croatia; ^6^ Einsten, Startup for Research, Development, Education, Trade and Services, Split, Croatia; ^7^ European Institute for Talents, Education, Research and Development, Split, Croatia; ^8^ Professore Emerito di Fisiologia, Università di Udine, Udine, Italy; ^9^ LaBiodin Biodynamics Laboratory, Universidade Federal do Rio Grande do Sul, Porto Alegre, Brazil

**Keywords:** running ecomomy, endurance runners, human locomotion, metabolic cost, team sport, talent detection & development

## Abstract

**Purpose:** In the last decades, the energy cost assessment provided new insight on shuttle or constant running as training modalities. No study, though, quantified the benefit of constant/shuttle running in soccer-players and runners. Therefore, the aim of this study was to clarify if marathon runners and soccer players present specific energy cost values related to their training experience performing constant and shuttle running.

**Methods:** To this aim, eight runners (age 34 ± 7.30y; training experience 5.70 ± 0.84y) and eight soccer-players (age 18.38 ± 0.52y; training experience 5.75 ± 1.84y) were assessed randomly for 6’ on shuttle-running or constant-running with 3 days of recovery in-between. For each condition, the blood lactate (BL) and the energy cost of constant (C_r_) and shuttle running (C_Sh_) was determined. To assess differences for metabolic demand in terms of C_r_, C_Sh_ and BL over the two running conditions on the two groups a MANOVA was used.

**Results:** $V^{\cdot }$ O_2max_ were 67.9 ± 4.5 and 56.8 ± 4.3 ml·min^−1^ kg^−1^ (*p* = 0.0002) for marathon runners and soccer players, respectively. On constant running, the runners had a lower C_r_ compared to soccer players (3.86 ± 0.16 J kg^−1^m^−1^ vs. 4.19 ± 0.26 J kg^−1^ m^−1^; F = 9.759, respectively; *p* = 0.007). On shuttle running, runners had a higher C_Sh_ compared to soccer players (8.66 ± 0.60 J kg^−1^ m^−1^ vs. 7.86 ± 0.51 J kg^−1^ m^−1^; F = 8.282, respectively; with *p* = 0.012). BL on constant running was lower in runners compared to soccer players (1.06 ± 0.07 mmol L^−1^ vs. 1.56 ± 0.42 mmol L^−1^, respectively; with *p* = 0.005). Conversely, BL on shuttle running was higher in runners compared to soccer players 7.99 ± 1.49 mmol L^−1^ vs. 6.04 ± 1.69 mmol L^−1^, respectively; with *p* = 0.028).

**Conclusion:** The energy cost optimization on constant or shuttle running is strictly related to the sport practiced.

## Introduction

The main physiological determinants of performance in endurance events are maximal oxygen uptake ( $V^{\cdot }$ O_2max_), energy cost (or running economy), and metabolic thresholds (Bassett and Howley, 2000; di Prampero, 2003). The energy cost is a critical determinant of endurance performance, mainly in homogeneous athlete groups (Conley and Krahenbuhl, 1980). On the other hand, the team-sport modalities differ from constant endurance events due to the intermittent characteristic (Stølen et al., 2005). In many team-sport modalities, the players need to perform short-duration sprints interspersed with low-intensity activities (Stølen et al., 2005). One critical feature is the ability to produce the best possible sprint performance over a series of sprints (Padulo et al., 2012; Padulo et al., 2015a; Padulo et al., 2015b; Padulo et al., 2016). The energy cost is an important determinant of performance in shuttle running exercise together with other indices (e.g., lactate threshold, oxygen kinetics, the velocity associated with $V^{\cdot }$ O_2max_ (Bishop et al., 2011).

Although there is a potential for transferring cardiorespiratory adaptations between different exercises, adaptive responses are limited by time and type of activity (McArdle et al., 1978; Basset and Boulay, 2000). Indeed, training programs including intermittent and constant running exercises have often been proposed. However, although the specific adaptations of these types of exercises are recognized, a comparison of energy cost responses between athletes in constant versus shuttle running modalities in the two conditions in a controlled study is still lacking. Also, while the age (Rittweger et al., 2009; Cho et al., 2021) and sex (Helgerud, 1994) are factors affecting the performance responses on shuttle and constant running, it is unclear which are the critical mechanisms underlying the different systems in our body, responsible for these differences. The weekly load of running is quite major in endurance runners (80–120 km per week) in comparison to soccer players (20–40 km per week) (di Prampero and Osgnach, 2018). In contrast, soccer players perform commonly sprints and shuttles while endurance runners rarely.

The energy cost represents the mass-specific energy expenditure per unit distance traveled taking into consideration the combustion enthalpy to oxidate the substrates used (Peyré-Tartaruga et al., 2021). The specificity of energy cost has been explored in terms of gradient and terrain types showing controversial findings. Although level, uphill and downhill running constitute biomechanically different modes of exercise (Padulo et al., 2013) economical runners on level surfaces are also economical on uphill and downhill grades (Breiner et al., 2019). Similarly, orienteer runners have similar energy cost between treadmill and path running (Jensen et al., 1994)^.^ Conversely, a previous study has found differences in the energy cost using athletes highly habituated to these specific conditions (e.g., orienteers/mountain versus track runners), showing lower values of energy cost to that condition where the athletes were trained (Jensen et al., 1999). Also, mechanical determinants of running performance seem to be specific to gradient (Padulo et al., 2013) and speed factors (Lemire et al., 2021), emphasizing the importance of test specificity in the performance evaluation in running. These conflicting findings challenge the specificity of the energy cost between exercises at constant-versus non-constant speeds. However, to the best of our knowledge, the response for this question is still unknown. Also, responses integrating aerobic and anaerobic components to energy cost through measurements of blood lactate concentration and oxygen uptake seem important to compare these different exercises. Previous studies have compared the oxygen consumption and blood lactate concentration at a fixed (relative to maximal) aerobic speed (Buchheit et al., 2011). However, these studies did not perform specific maximal tests considering the constant and non-constant conditions and, therefore, hampering adequate comparison at same metabolic intensities (Ardigò et al., 2020).

We aimed to compare the total energy cost of running in constant and shuttle conditions between athletes habituated to constant run or with changes of direction, respectively. We hypothesized that athletes will be more economical in the specific mode of exercise. Furthermore, we expect that athletes habituated to shuttle running will have the highest lactate concentration in the shuttle running condition due to higher anaerobic capacity in soccer players than endurance runners (Bangsbo et al., 1993). These possible differences may be related to cell metabolism, and specifically to functional characteristics related to muscle fiber type (Bangsbo et al., 1993; Hopwood et al., 2023).

## Material and methods

### Participants

Sixteen participants, eight marathon runners [age 34.00 ± 7.3 years (range 24 < 40y), body mass 65.13 ± 6.53 kg, stature 1.73 ± 0.06 m, body mass index 21.67 ± 1.55 kg·m^−2^, training experience 5.70 ± 0.84 years] and eight soccer players [18.38 ± 0.52 years (range 18 < 19y), body mass 71.38 ± 7.07 kg, stature 1.76 ± 0.06 m, body mass index 23.01 ± 1.53 kg·m^−2^, training experience 5.75 ± 1.84 years] voluntarily participated in this study. Inclusion criteria were >80 km per week and personal best on marathon race <2 h 40’ in the last 6 months (for runners), Second Team in Serie A League for soccer players, >4 training sessions per week (for runners and soccer players) and the training experience of ∼5 years for both groups. Subjects were healthy with no muscular, neurological, or tendinous injuries. The study was carried out following the recommendations of the Code of Ethics of the World Medical Association and approved by the local University. All subjects gave written informed consent following the Declaration of Helsinki and were free to withdraw from the study at any time.

### Experimental design

This investigation was performed on three single days. On the first day, each participant was assessed on $V^{\cdot }$ O_2max_ test on a calibrated treadmill (Technogym™ Excite 900, Gambettola, Italy). After a 7-min warm-up of running at 8 km·h^−1^ and 5-min of passive recovery for each participant the $V^{\cdot }$ O_2max_ test started running with at 8 km·h^−1^ and increased by 1 km·h^−1^ every 1 min until voluntary exhaustion. After 4 days of $V^{\cdot }$ O_2max_ test each participant was assessed randomly on constant or shuttle running and inverted (shuttle or constant running) after 3 days, so each participant completed all running conditions.

Constant running test was performed on a calibrated treadmill (previously used). Each participant after a 7-min warm-up of running at 8 km·h^−1^ and a 5-min of passive recovery, performed a 7-min test of running at 10 and 12 km·h^−1^ for soccer players and marathon runners, respectively {corresponding to $V^{\cdot }$ O_2max_ ∼60% [Typical intensity for runners (Davies and Thompson, 1979) previously calculated from the $V^{\cdot }$ O_2max_ test ( $V^{\cdot }$ O_2_-60) for both groups]}.

Shuttle running test was performed on rubber surface (5 mm) in an indoor gym. Each participant after a 7-min warm-up of running at 8 km·h^−1^ on a treadmill (previously used) and a 5-min of passive recovery, performed on 7-min shuttle run with a changes of direction (180°) every 5 s (20 s of shuttle run and 20 s of passive recovery, repeated for 11 times) according to Buglione and di Prampero (2013).

The distance and the speed for shuttle run in each group was previously estimated (Buglione and di Prampero, 2013) to obtain the 80% $V^{\cdot }$ O_2max_ [typical intensity for team sport (Stølen et al., 2005) during the match ( $V^{\cdot }$ O_2_-80)] resulted of 22 m (15.84 km·h^−1^) and 20 m (14.40 km·h^−1^) in 5 s, for marathon runners and soccer players, respectively. The speed was set by computer-driven loudspeakers, emitting beeps the frequency of which was programmed in such a way that, to achieve a given known average speed, the runner at the beep had to be precisely at the level of a cone placed laterally on the track. All shuttle runs were carried out on a linear path. Throughout each run, the subject accelerated and decelerated as required to maintain the average speed.

Energy cost of constant running (C_r_) and shuttle running (C_Sh_) was calculated as the ratio between the metabolic energy expenditure above resting (assumed = 3.5 mL·kg^−1^·min^−1^) measured in ml O2 and converted in J (assumed as 1 mL of O_2_ = 20.9 J·ml^−1^ which is strictly correct for an RQ = 0.96) and the speed (in m·s^−1^) as done by Buglione and di Prampero (2013) ^.^ The lactic contribution to the overall energy expenditure (AnLa) was estimated from the net [La] accumulation after exercise, above resting (assumed = 1 mM), based on an energy equivalent of [La] accumulation in blood of 3 mL O_2_·kg^−1^·mM^−1^ (di Prampero, 1981). Oxygen consumption was measured on a breath-by-breath basis using a portable metabograph (K4b^2^, Cosmed, Rome, Italy; oxygen consumption technical error of measurement 0.14 l·min^−1^, intra-class correlation coefficient 0.85. Portable unit was calibrated for flows using a 3-l syringe and for gasses percentages using a mixture of known composition (16% O_2_ and 5% CO_2_ in N_2_). During tests, portable unit was kept in a knapsack on subject’s shoulders. Data were recorded and telemetrically sent to a personal computer by portable unit. Oxygen consumption data were averaged over 30-s windows. Oxygen consumption was measured continuously during constant/shuttle running and 6-s during the passive recovery (Buglione and di Prampero, 2013) to quantify the O_2_ debt payment for C_Sh_. Blood lactate concentration [(La)] was determined (BLa-Constant and BLa-Shuttle) using a portable lactate analyzer (Lactate Pro LT 1710; Arkray Inc., Kyoto, Japan) on a blood sample obtained from the ear lobe at the very end of tests or 3 min after the end of it. The energy cost was also estimated using the approach proposed by di Prampero and Osgnach (2018). The analysis procedures and results are presented in Supplementary Material S1.

### Statistical analysis

Statistical analysis was performed using JASP software (Amsterdam, Netherlands). Results were expressed as mean ± standard deviation (SD). Shapiro-Wilk test was used to test assumption of normality of both dependent and independent variables. To assess differences for metabolic demand in terms of C_r_, C_Sh_ and BL over the two running conditions (constant running—shuttle running) on the two groups (Marathon runners—Soccer players) a MANOVA was used. Assumption of equality of covariance matrices was tested with Box’s M test—a statistically non-significant value is preferred (*p* > 0.05 ns). The Hotelling-Lawley Test (*H* ^2^) was used to test the multivariate effect of variables. Moreover, the effect size of differences between groups—across dependent variables—was assessed with the eta-squared (η^2^) and its benchmarks. Rejection level was set at *α* < 0.05.

Moreover, considering the small sample size of each group (*n*1 = *n*2 = 8), classic inferential analyses (i.e., *t*-test) were complemented by Bayesian *t*-tests to assess differences between groups. In detail, independent sample *t-*tests were performed to assess mean comparisons for: anthropometric measurements (age, body mass, body height, body mass index, and training experience) and physiological measurements ( $V^{\cdot }$ O_2max_, $V^{\cdot }$ O_2_-60, $V^{\cdot }$ O_2_-80, C_r_, C_Sh_, BLa-Constant, BLa-Shuttle). More in detail, for Bayesian statistics, the JASP’s default value was used to set the prior distribution: thus, a zero-centered Cauchy distribution with a default scale – γ (width parameter)—of 0.707: [δ ∼ Cauchy (0, 0.707)]. Evidence for the alternative hypothesis (H_1_) was observed by means of the Bayes Factor (BF). According to the Jeffery’s scheme (Jeffreys, 1961) BF_10_ values (as effect sizes) can be considered as “anecdotal” (1 < BF < 3), “moderate” (3 < BF < 10), “strong” (10 < BF < 30), “very strong” (30 < BF < 100), or “extreme” (BF > 100) relative evidence for a hypothesis (H_0_ or H_1_).

## Results

The anthropometrics and training background data are not significantly differently with *p* > 0.05 for body mass with a BF_10_ equal to 1.237 (anecdotal), body height with a BF_10_ equal to 0.592, body mass index with a BF_10_ equal to 1.115 (anecdotal), training experience with a BF_10_ equal to 0.429 (anecdotal) except for age with a BF_10_ equal to 5.010 (moderate) with *p* = 0.003.

**FIGURE 1:**
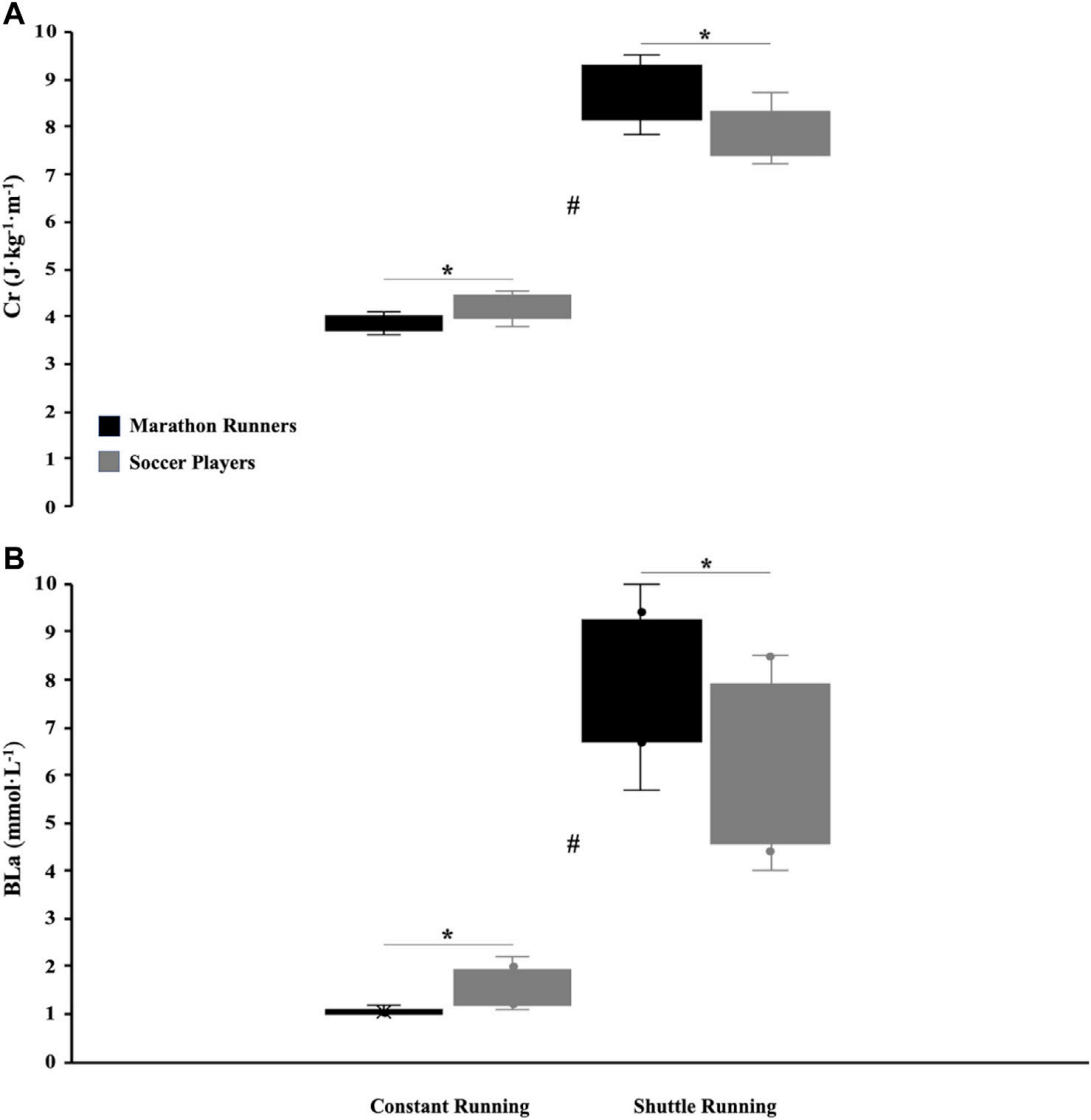
Energy cost **(A)** and blood lactate **(B)** in-between running conditions (^#^ *p* < 0.05) and between soccer players and marathon runners (*p < 0.05).

The physiological measurements showed a $V^{\cdot }$ O_2max_ of 67.91 ± 4.50 reached at 22.00 ± 1.22 km·h^−1^ and 56.75 ± 4.27 ml·min^−1^ kg^−1^ reached at 18.22 ± 1.39 km·h^−1^ [BF_10_ equal to 125 (very strong) with *p* = 0.0002] for marathon runners and soccer players, respectively. The $V^{\cdot }$ O_2_-60 on constant running was 59.37 ± 9.07 and 60.93 ± 5.89% V̇O_2max_ [BF_10_ equal to 0.453 (anecdotal) with *p* > 0.05] with a $V^{\cdot }$ O_2_ of 40.17 ± 5.75 and 34.36 ± 0.95 ml·min^−1^ kg^−1^ for marathon runners and soccer players, respectively. The $V^{\cdot }$ O_2_-80 on shuttle running was 80.36 ± 5.45 and 80.54 ± 3.97% $V^{\cdot }$ O_2max_ [BF_10_ equal to 0.428 (anecdotal) with *p* > 0.05] with a $V^{\cdot }$ O_2_ of 50.46 ± 3.54 and 45.67 ± 3.72 ml·min^−1^ kg^−1^ for marathon runners and soccer players, respectively. MANOVA assumptions for energy cost were respected: Box’s M Test: χ^2^ = 1.370; *y* = 0.713 and Shapiro Wilk test = 0.977 *p* = 0.940. The energy cost was affected by (group or condition) (Figure 1A) for both groups (marathon runner and soccer players) for each running condition (constant running—shuttle running) was significantly different (main effect: H-L = 1.682; F = 10.932, *p* = 0.002). On constant running (C_r_) the marathon runners had a lower C_r_ (3.86 ± 0.16 J·kg^−1^·m^−1^) minus 8% compared to the soccer players [4.19 ± 0.26 J·kg^−1^·m^−1^; F = 9.759 with *p* = 0.007; with a BF_10_ equal to 12.697 (strong)] while on shuttle running the marathon runners had a higher C_Sh_ (8.66 ± 0.60 J·kg^−1^·m^−1^) plus 10% compared to the soccer players [7.86 ± 0.51 J·kg^−1^·m^−1^; F = 8.282 with *p* = 0.012, with a BF_10_ equal to 8.112 (moderate)]. MANOVA assumptions for BLa were not respected: Box’s M Test: χ^2^ = 12.792; *p* = 0.005 and Shapiro Wilk test = 0.9812 *p* = 0.004. However, considering that MANOVA is quite robust to violation of assumption, the statistic was still performed (Tabachnick and Fidell, 2013; Milavic et al., 2019). The same metabolic responses of C_r_/C_Sh_ there was on BLa (Figure 1B) for both groups (marathon runners and soccer players) for each running condition (constant running—shuttle running) showing significant differences (H-L = 1.108; F = 7.200, *p* = 0.008). BLa-Constant running was very lower (32%) for marathon runners (1.06 ± 0.07 mmol·L^−1^) compared to the soccer players [1.56 ± 0.42 mmol·L^−1^; F = 11.133 with *p* = 0.005, BF_10_ equal to 8.749 (moderate)], conversely the BLa-Shuttle for marathon runners was 7.99 ± 1.49 mmol·L^−1^ very higher (32%) compared to the soccer players [6.04 ± 1.69 mmol·L^−1^; F = 5.973 with *p* = 0.028, BF_10_ equal to 2.539 (anecdotal)].

## Discussion

There is growing evidence that energy cost is affected by the specificity of movement. Notably, the constant and intermittent pace is markedly related to the type of mechanical work produced and respective mechanical efficiencies (Zamparo et al., 2016; Zagatto et al., 2023). However, little data exist on running energy costs between these conditions and among athletes trained especially for these conditions. Therefore, the present study compared energy costs between constant and intermittent runs in two groups, each highly trained for one of these events. Thus, we assessed the differences on energy cost between these two athlete groups under constant versus shuttle conditions. Overall, we observed that energy cost of running was specific to training background of athletes analyzed, confirming our first hypothesis. Marathoners were more economical than soccer players at constant conditions, and soccer players spent less metabolic energy than marathonists in shuttle conditions. Furthermore, this specificity of energy cost of running was similarly represented by the aerobic and anaerobic components of exercise. Thus, our second hypothesis was rejected, showing that under these submaximal intensities (60%–80% of $V^{\cdot }$ O_2max_) the blood lactate concentration was lower for athletes habituated to these conditions similarly to the findings from the aerobic component of energy cost.

It should also be pointed out that the higher energy cost of shuttle running in marathoners as compared to soccer players is partly due to the greater acceleration and deceleration observed in the former, because of the slightly longer distance (22 vs. 20 m) covered at the same time (5 s) over each shuttle run. However, as discussed in some detail in the Supplementary Material S1 the acceleration/deceleration effect is not enough to justify entirely the larger energy cost of shuttle running of the marathoners, thus supporting the view that the soccer players are indeed more economical in this type of exercise. The study used athletes at different ages and therefore this factor should be pointed out. Both the energy cost (Pantoja et al., 2016) and the main mechanical determinants of the energy cost of running (Cavagna et al., 2008) worsen with age. In addition to the crossover design, our study suggests a possible effect of age on outcomes, and the main messages of the study, i.e., about specific training effects on energy cost responses were maintained.

The present study confirms the importance of training specificity about energy cost of running adaptations (Lemire et al., 2021). In addition, it reinforces the need for specific evaluations for the type of exercise that comprises the sport modality. Furthermore, the present study shows the importance of evaluating physiological determinants of performance in athletes trained for specific testing conditions, revealing a degree of specificity of the energy cost of running that had not previously been found due to the use of non-specific groups of athletes (Jensen et al., 1994; Breiner et al., 2019)^.^ Even, interestingly, our findings show that the energy cost of running seems to be a crucial factor and subject to important adaptation in athletes who perform intermittent activities such as soccer. This finding opens the question about a possible role of the energy cost of running performed under intermittent conditions on overall performance in team sports. Further, we confirm the findings on $V^{\cdot }$ O_2max_ from previous studies (McArdle et al., 1978; Basset and Boulay, 2000), and extend our understanding of specificity for this important parameter in steady state versus intermittent condition of exercise.

Furthermore, these observations could explain the controversial on energy cost from previous studies (Buchheit et al., 2011; Bekraoui et al., 2020). When the characteristics of runners are related to running task, the cardiorespiratory and metabolic results are clearly different between distance runners and soccer players. The calculation of the energy expenditure per distance unit (energy cost) permits us to observe that for elite distance runners, running at non-constant speed is physiologically non-optimal, while at habitual mode of running for them, at constant speed, is physiologically optimal. For soccer players, these specifics occurred in exactly the opposite mode.

The lactate concentration results demonstrate that the aerobic and anaerobic components similarly interfere with the cost of moving through the terrain, resulting in impactful differences in overall exercise metabolism between marathon runners and soccer players. Most interestingly, elevated lactate levels indicate that even highly trained athletes have negative metabolic outcomes when they are required to perform activities at a pace/mode they have not been trained (Margaria et al., 1964; Bangsbo et al., 1993). Further, the results of the energy cost of running are in line with previous findings in distance runners for constant conditions (Ardigò et al., 2023) and at shuttle condition (Zamparo et al., 2016).

Energy cost is the needed quantity of metabolic energy to displace the body per unit of distance and mass (Peyré-Tartaruga et al., 2021). The reasons for these specific responses of energy cost between marathon runners and soccer players are following, 1) fiber type and cell oxidative function; and 2) type of mechanical work production. Previous findings have shown that the relative number of type IIx muscle fibers was lower and the expression of the monocarboxylate transporter 1 was higher after a 5-week speed endurance training (Gunnarsson et al., 2012). Interestingly, the energy cost of running was evaluated just at 10 km·h^−1^ at constant conditions. Probably the difference would be more impactful if evaluated at non-stable conditions. Furthermore, the differences in energy cost may be attributable to the type of muscle-tendon work that is performed during runs at constant and non-constant speed. While at constant speed the muscle-tendon units perform equally positive and negative work during one complete step, during acceleration and braking, the work is predominantly positive and negative, respectively (Zamparo et al., 2016). An important repercussion is that the metabolism operates at very different mechanical efficiencies at different stages of a shuttle run. The energy expenditure to perform braking is much lower than to perform acceleration, therefore indicating the metabolism to a much higher requirement oscillation condition than at constant speed running (Buglione and di Prampero, 2013). Another physiomechanical repercussion is that in short shuttles the elastic mechanism of minimizing energy expenditure is reduced compared to constant running, but this difference decreases as the shuttle distance increases (Buglione and di Prampero, 2013; Zamparo et al., 2016). Recently, the energy cost of running was related to the production of tendon work (Monte et al., 2020). Thus, probably specific adaptations in the task of storing and reusing elastic energy are specific to the type of task and may justify, at least partially, the responses found in the present study (one comprehensive review of these adjustments may be found in Peyré-Tartaruga et al., 2021). The muscle fiber characteristics, therefore, should partly explain for the specific metabolic economy observed here. It has been shown that soccer players have a higher percentage of type IIx muscle fibers than endurance runners (Bangsbo et al., 1993; Gunnarsson et al., 2012) due to repetitive actions of accelerations, decelerations and change-of-directions.

In conclusion, the results of the present study clearly demonstrate the specificity of the energy cost of running to type of exercise performed (constant versus shuttle) and consequently to training background for distance runners and soccer players. This study reinforces the importance of specificity to training adaptations, showing the necessity to test an athlete while performing the activity they are trained for. Other strength of this study was comparing two different groups of athletes shading light on performance determinants in a comparative perspective. Furthermore, this study brings new insights into talent detection and development topics and highlights the significance of evaluating the physiological factors impacting performance in athletes who have undergone specific training. One possible use of the present findings is about running cross-training models throughout the pre-season and throughout the season. Distance runners can use shuttle training methods (e.g., HIIT) as preparation for cross-country races where changes in speed and race pace is common. On the other hand, constant training at moderate intensity can be useful in pre-season, in micro cycles and regenerative sessions (e.g., post-match), and in injury recovery phases. This research could shed new light on how different muscle fiber types contribute to the energy cost and running economy in these athletes and how it affects their performance. This information also could be useful in talent development process, as the knowledge which can be used to optimize training and performance. There are some limitations in the current study, as the gender (male), as for this type of investigation, it would be more useful to investigate both male and female athletes. Second, the groups have different ages. Similar age groups related to the same training experience could better clarify the physiological adaptations, although in our study, the time experience in the sports was balanced.

An interesting future research direction could be to investigate the role of muscle (metabolism, muscle fiber type, pulmonary function) and transmission (co-contraction, muscle sequencing, joint coordination) efficiency aspects (Peyré-Tartaruga and Coertjens, 2018) in the energy cost optimization during constant or shuttle running in both marathon runners and soccer players. Additionally, conducting research on a sample of twins to determine the influence of the environment in relation to genetic inheritance would also be of interest.

## Data Availability

The original contributions presented in the study are included in the article/Supplementary Material, further inquiries can be directed to the corresponding author.
