# Supplementary material for: Energy cost differences between marathon runners and soccer players: Constant versus shuttle running
Source: Front Physiol. 2023 May 3;14:1159228. doi: 10.3389/fphys.2023.1159228 (PMC10206428; doi:10.3389/fphys.2023.1159228)
Supplement: Supplementary file 1 [file Table1.pdf]

Supplementary material 01 of 'Energy cost differences between marathon runners and soccer players: constant versus shuttle running.

In the "shuttle runs" the athlete's speed at the beginning and end of each phase is equal to 0 and, as shown by Zamparo et al. (2019) for distances of 5, 10, 15, and 20 meters, the time course of the speed over each phase follows an essentially sinusoidal time course. Furthermore, these authors have also shown that, for distances of 20 m covered at maximal speed, the ratio between peak and mean speed over each phase is ≈ 1.4. It follows that in the initial phase of each run the subject accelerates up to a maximum speed, whereas in the final phase he decelerates from this same speed to zero. Therefore, since for soccer players and marathon runners the distance was 20 and 22 m, covered in all cases in 5 seconds, the appropriate peak speeds can be estimated from the know average values as 5.60 and 6.16 $m \cdot s^{-1}$, respectively.

As shown by di Prampero et al. (2005), from a biomechanical point of view, accelerated running on flat terrain is analogous to uphill running at constant speed, where the slope is proportional to the acceleration. Likewise, decelerated running on flat terrain is analogous to downhill running at constant speed, where the slope is proportional to the deceleration. Hence, the slope that makes the energy cost of accelerated (or decelerated) running on flat terrain equal to that of running uphill (or downhill) at constant speed was defined "Equivalent Slope" (ES). Furthermore, since during accelerated or decelerated running the force that must be applied along the subject's head to feet axis is slightly higher as compared to that applying at constant speed, di Prampero et al. (2005) defined "Equivalent Mass" (EM), the increase in weight that would make the run during acceleration or deceleration equal to that uphill or downhill at constant speed at the slope in question. Finally, di Prampero et al. (2005) have shown that: i) ES is equal to the ratio of the forward acceleration or deceleration (± a) to the acceleration of gravity ($g = 9.81\ m \cdot s^{-2}$), ES =

±a/g; and: ii) EM = √(±ES^2+1). Therefore, knowledge of the time course of the speed and acceleration during accelerated or decelerated running on flat terrain allows one to calculate the corresponding ES and EM values and hence to estimate the corresponding instantaneous energy cost. Indeed, the energy cost of running at constant speed uphill or downhill is well known (e.g., see Minetti et al. (2002)).

As mentioned above, this approach allows one to estimate the instantaneous values of the energy cost of accelerated and decelerated running on flat terrain if provided that the time course of speed and acceleration are known. This is not always the case, nor was it in this specific instance. However, knowledge of the mean and peak speed allowed us to estimate the overall energy cost of shuttle running, on the basis of a simplified procedure, describe in detail elsewhere by di Prampero and Osgnach (2018).

More specifically, in this study the instantaneous energy cost throughout the 100 m world record by Usain Bolt (9.58 s, Berlin, 2008) was assessed on the basis of the appropriate time courses of velocity and acceleration. The time integral of the so obtained instantaneous energy cost yielded the overall energy expenditure to cover the entire 100 m distance: it amounted to 0.757 kJ·kg^{-1}. The overall energy expenditure was subsequently estimated assuming that the additional energy spent in the acceleration phase (E_{acc}), over and above that for constant speed running, is described by:

$$E_{acc} = M\ v_{peak}^2/(2\ \eta_{pos}) \qquad \text{i)}$$

where M is the subject's body mass, v_{peak} the peak speed, and η_{pos} the efficiency of converting metabolic into mechanical energy during the acceleration phase. Thus, expressing per unit body mas and assuming η= 0.25 (Cavagna et al., 1971; Cavagna and Kaneko, 1977), equation i) can be rewritten as:

$$E_{acc}/M = v_{peak}^2/0.5 = E_{acc} \qquad \text{ii)}$$

If this is so, the overall amount of energy spent to cover any given distance (d) from a still start, per unit body mass, is given by:

$$E_{tot} = (C_0 + k\ v_{mean}^2)\ d + E_{acc} = (C_0 + k\ v_{mean}^2)\ d + v_{peak}^2/0.5 \qquad \text{iii)}$$

where C_0 is the energy cost of running at constant speed, v_{mean} the mean speed, and $k\ v_{mean}^2$ the amount of energy spent to overcome the air resistance.

Assuming $C_0 = 3.8$ J·(kg m)$^{-1}$, as can be expected for a top level runner, k = 0.01 J s^2·(kg m^3)$^{-1}$ and the mean and peak speeds as observed for Bolt extant world record (10.44 and 12.35 m·s^{-1}, respectively) E_{tot}, as calculated according to equation iii) turns out to be 0.794 kJ·kg^{-1}, i.e. rather close to the value obtained from the time integral of the instantaneous metabolic power which, as mentioned above amounted to 0.757 kJ·kg^{-1} (di Prampero and Osgnach, 2018). This approach was extended to include also the extant 200 m world record by Usain Bolt (19.19 s) and a top 400 m performance by LaShawn Merrit (44.06 s) yielding values between 89 and 99 % of those obtained from the time integral of the corresponding instantaneous metabolic power (di Prampero and Osgnach, 2018). It can be concluded that the simplified approach describe by equation iii) yields rather accurate estimates of the overall energy expenditure over short sprint distances.

However, when this simplified approach is applied to shuttle running, as in this specific instance, the energy cost of the deceleration at the end of each phase (from v_{peak} to zero) must also be considered. This can be estimated as from equation i), assuming that the efficiency of converting metabolic into mechanical energy during the deceleration phase (η_{neg}) is about – 1.0, as shown by several authors (e.g., see Margaria, (1938) and Minetti et al. (2002)) for the expected range of Equivalent Slopes.

Hence:

$$E_{dec} = - M\, v_{peak}^2/(2 \times \eta_{pneg}) \qquad \text{iv)}$$

wherefrom, assuming (η_{neg}) = -1.0 , and since during the deceleration phase the mechanical work is negative, and we obtain:

$$E_{acc}/M = v_{peak}^2/2.0 = E_{dec} \qquad \text{v)}$$

Hence equation iii) must be modified as follows:

$$E_{tot} = (C_0 + k\, v_{mean}^2)\, d + E_{acc} + E_{dec} =$$

$$= (C_0 + k\, v_{mean}^2)\, d + v_{peak}^2/0.5 + v_{peak}^2/2.0 = v_{peak}^2 \times 2.5 \qquad \text{vi)}$$

The E_{tot} values obtained from this equation based on the appropriate average and peak speeds were subsequently normalized per unit of distance to estimate the energy cost of shuttle running ($C_{\mathbf{sh\text{-}est}}$, J·(kg m)$^{-1}$for both marathon runners and soccer players. These values are reported in Table I, together with the directly measured ones ($C_{sh\text{-}meas}$), with the ratio between estimated and directly measured values ($C_{sh\text{-}est}/C_{sh\text{-}meas}$), and with the energy cost of running at constant speed (C_0).

As shown in Table I, the ratio between the energy cost of shuttle running, as estimated thanks to equation vi, and the directly measured values is rather close to 1.0. Hence this equation allows one to obtain a sufficiently accurate estimate of the energy cost of accelerated and decelerated running events, provided that the peak and average speeds thereof are available.

Table I. Average values of the energy cost of running ($J \cdot kg^{-1} \cdot m^{-1}$) directly determined at constant speed (**C_0**) or over 20 and 22 m shuttle running (**$C_{sh-meas}$**) for marathon runners and soccer players. The energy cost of shuttle running estimated from equation vi) (**C_{sh-est}**) is also reported (see text for details) together with the ratio **C_{sh-est} C/$_{sh-meas}$**.

Groups	C_0	$C_{sh-meas}$	C_{sh-est},	$C_{sh-est}/C_{sh-meas}$
Marathoners	3.86	8.66	8.37	0.967
Soccer player	4.19	7.86	7.63	0.971

In conclusion, even if the marathoners are substantially more economical than soccer players when running at constant speed, for both groups the average cost during shuttle running increase in direct proportion with the peak speed and can be estimated rather accurately from the simplified approach described above.

References

Cavagna, G. A., and Kaneko, M. (1977). Mechanical work and efficiency in level walking and running. *J. Physiol.* 268, 467–481. doi: 10.1113/jphysiol.1977.sp011866.

Cavagna, G. A., Komarek, L., and Mazzoleni, S. (1971). The mechanics of sprint running. *J. Physiol.* 217, 709–721. doi: 10.1113/JPHYSIOL.1971.SP009595.

di Prampero, P. E., Fusi, S., Sepulcri, L., Morin, J. B., Belli, A., and Antonutto, G. (2005). Sprint running: A new energetic approach. *J. Exp. Biol.* 208, 2809–2816. doi: 10.1242/jeb.01700.

di Prampero, P. E., and Osgnach, C. (2018). Metabolic Power in Team Sports - Part 1: An Update. *Int. J. Sports Med.* 39, 581–587. doi: 10.1055/a-0592-7660.

Margaria, R. (1938). Sulla fisiologia e specialmente sul consumo energetico della marcia e della corsa a varia velocità ed inclinazione del terreno. *Atti Accad. Naz. dei Lincei 7*, 299–368.

Minetti, A. E., Moia, C., Roi, G. S., Susta, D., and Ferretti, G. (2002). Energy cost of walking and running at extreme uphill and downhill slopes. *J. Appl. Physiol.* 93, 1039–1046. doi: 10.1152/japplphysiol.01177.2001.

Zamparo, P., Pavei, G., Monte, A., Nardello, F., Otsu, T., Numazu, N., et al. (2019). Mechanical work in shuttle running as a function of speed and distance: Implications for power and efficiency. *Hum. Mov. Sci.* 66, 487–496. doi: 10.1016/j.humov.2019.06.005.
